# Address

**Published:** 1889-12

**Authors:** C. M. Wright


### THE DENTAL REGISTER.

VOL. XLIII.] DECEMBER, 1889. [No. 12.

### COMMUNICATIONS.

### Address.

BY THE PRESIDENT, PROF. C. M. WRIGHT, D.D.S.

Before the Ohio State Dental Society, at Cleveland, Oct. 29th, 1889.

*Gentlemen, Members of the Ohio State Dental Society:*

By invitation of your Executive Committee I have been placed in the position of a precedent breaker; for several of my distinguished predecessors have declined to offer an annual "address"—a presidential address, upon these occasions. And they were wise.

I felt so deeply the compliment paid me by the committee—my bosom was in such a hypertrophied condition with the pride which filled me to the brim, on account of the honor which I know has always surrounded this chair—my internal elation was so great when I thought of the dignity of this position, which the kind indulgence of the society permitted me to grasp this year—that my usual modesty and ordinary judgment forsook me, and I agreed to deliver an address before this honorable and dignified body to-day. An *address* is a stately affair and should abound in wisdom. An *address* should be a dignified oration, historical and advisory in its character. In it, should be presented comparisons of the past and present conditions of an organization, a report of the progress of the organization, or of a profession, in science, in education, in social, and other relations. An address before a State Society (the most august association of a large territory) should present in detail the movements of the entire profession of that State in its literary, its legal, and its scientific activity, at least during the past year of its existence. This, no doubt, is what was expected of me when your honorable committee tendered me the invitation. But, this is just what I do not feel capable of accomplishing in a satisfactory manner. After much thought upon the subject I went to my friends in consultation, and but one subject was offered (and this by a valued friend) which it was believed would be of interest and value to offer for your consideration; that subject was in relation to the Dental Law of the State. Its present condition, and the necessity for action on the part of this body, this winter, if we would make it more effectual, should be a matter for consideration. The law as it now exists is imperfect in some respects. Other states have much more efficient laws, and complaints are heard all over our State from members of the profession in regard to the laxity of restraint placed upon practice, and against the influx of incompetent and unqualified men who are crowding into our State, driven from the more rigid neighboring states; and this to the detriment of our position as a profession at home, and to our reputation among other states, as well as in foreign countries. Thus, as a matter of pure self-defense, prompt action should be taken in the matter, and if it is found either impractical or impossible to obtain from our legislators an entirely new law, as was tried last winter—and this I may add is not held as a real necessity—a vigorous and intelligent effort to effect such amendments to the existing law as shall make it more in accord with the demands of the times, and more effective in its practical workings, should be made.

This, it is believed, can be done this winter. These amendments should include a "registration act," which alone, if well adjusted, might be made to revivify a law which now seems to be a "dead letter." To do this is the duty of a State Society. To do this promptly and well is the duty of the hour. To do this properly and effectively is the duty of a competent committee appointed by this society, and having power to act freely for this society, and to be compensated by this society, if compensation is necessary in any way to secure the desired object.

It would not be appropriate, I am sure, gentlemen, for me at this time to go more fully into this matter, or to attempt to analyze the law as it stands, or offer advice on the details of the required amendments. Action--immediate action, is the present necessity, if our great State is not to be left behind in the general advancement. This State, gentlemen, which has given to the profession such men as Chapin A. Harris, James Taylor, and a host of others who have distinguished themselves in the past as the founders of the dental profession in America--we of Ohio, can not afford by any neglect on our part, to let the work of these men crumble into ruins. Ohio must not be the only state whose dental garden, if I may be permitted such an illustration, is allowed to be overrun with weeds. Weeds plucked up by the roots by the careful gardeners of other states, and thrown over the wall into our fair land, where they take root and flourish, to the infinite injury of our flower beds and lawns.

Another question of the day, which I shall take the liberty of referring to briefly, the suggestion of which presented itself to my mind in the discussion at a recent meeting of the Seventh District Dental Society of the State of Ohio, on the medical status of the dentist, and also from an editorial in the October number of the *Dental Review*, in which a dentist, or one who claims that he is only a dentist, has isolated and described a prevalent disease, which he, the writer, claims is epidemic in the ranks of the dental profession, and which he classifies as "M.D.--ism." The effect of the disease, according to this writer, are serious. It threatens to cramp the usefulness of the dental society, the dental college and the dental profession. It is claimed to be steadily on the increase in the east and west. The ætiology of the disease is still shrouded in some mystery, but an hypothesis can be constructed upon modern methods. A medical micro-organism, whose exact place in the microbe kingdom is not yet definitely established, has found a lodgement in the gray matter of the dentist's nervous tissue, where it finds pabulum and consequently opportunities for growth, development, and for reproduction of its kind according to the laws of all living things. In the assimilation of this pabulum, and the metabolic changes which occur, a ptomaine results which affects the habitat or gray tissue in which it is lodged, and the function of this important tissue is seriously interfered with—hallucinations are produced—things which before seemed right, now appear wrong; positions which before were considered honorable, now are regarded with contempt and abhorrence; names—such as dentists, or titles such as doctor of Dental Surgery—which, in the normal condition of the patient and before the effects of the micro-organism could be observed, were looked upon as creditable, now cause wave-like shudders to pass throughout the muscle tissue of the entire organism, the emotional symptom seeming to be a reflex nervous act, as from an opprobrious epithet applied to the patient, acting upon the terminal of a sensitive nerve. These are a few of the more prominent and characteristic symptoms. Have you studied the disease, gentlemen? And if so, have you evolved from the depths of your therapeutic knowledge a method of treatment?

That it is desirable to cure a disease of this character, I think is evident to every one of us. Dentistry is a well-known, a reputable, and a distinct profession. It has a history, a literature, and a system of special education of its own. We have, I sincerely believe, every reason to feel proud of each of these departments, and, as I have expressed before in public and in private, personally I have no sympathy with the false pride too prevalent now in our ranks, which makes a dentist, or a dentist's wife and daughters and sons sneer at the profession or at the name of the profession which has made him and them what they are. There are too many scholars in our ranks, too many scientists in our midsts for any man to feel ashamed to be ranked simply as a dentist. We have been lifted out of the mire by the efforts of noble men, whose example we want to emulate. And, if we want to look over our geneological tree, while its roots may have been planted in the medical orchard, the grafting has been so successful and the fruit of the branch of to-day is so fine that no man need reject it for the fruit of the rest of the tree.

Let Ohio dentists remain dentists and simply do the best they can to maintain the dignity, the respectability and the gentility of the profession of their choice. Every unit of the profession has this responsibility upon his shoulders. He can not shirk them. Every noble act of his reflects honor upon the entire profession. Every ignoble act of his, trivial as it may seem in the abstract, reflects dishonor upon the entire profession. No man can live for himself after he enters a profession. He may try to, but he can not do it. If you gain wealth, social position, literary distinction, wide reputation for any good thing, I benefit by it my brother, try as you may to keep me from it. If you lose caste socially, if you are envious and mean, if you resort to low methods of conducting yourself or business, brother, I suffer for it, and I can not escape it. We are so bound up into one tissue, so interdependent one upon another that our organization can be easily likened to any complex organism, where nerve and blood, and muscle, and gland, and bone seem only constructed for the uses of the whole and no one can be independent of the other.

Now, gentlemen, I have finished the address, which I fear partakes somewhat of the character of a sermon without a text. There are some other things which might, I am sure, be said which would be appropriate on an occasion like this, but I wanted to insert the soul of wit in this address and I can not do it if I prolong it. I thank you for your kind attention.

